# Supplementary material for: Changes in life expectancy and life span equality during the COVID-19 epidemic in 2020-22 in Japan
Source: PLoS One. 2026 Apr 29;21(4):e0345579. doi: 10.1371/journal.pone.0345579 (PMC13134763; doi:10.1371/journal.pone.0345579)
Supplement: S1 Methods — (DOCX) [file pone.0345579.s002.docx]

**S1 Methods: Life table calculation in the present study.**

Life table calculation in the present study followed standard techniques as described in conventional textbooks and in our previous study.[1–4] Briefly, we first calculated age group-specific mortality $m_{x}$ using death counts and exposure-to-risk population provided by JMD in each age group $x$. Using $m_{x}$ and $a_{x}$, the average length of time to death in deceased individuals in age group $x$, we calculated $q_{x}$, the probability of death in age group $x$ as:

$$q_{x}=\left\{ \begin{aligned} \frac{m_{x} w_{x}}{1+ \left( w_{x}-a_{x} \right) m_{x}}, x=0, 1-4, 5-9, \ldots95-99, \\ 1, x=100+, \end{aligned} \right.$$

where $w_{x}$ is the time interval of age group $x$. Starting from an initial population $l_{0}=100,000,$the survivorship $l_{x}$ at age $x$ can be obtained by iteratively applying the formula:

$$l_{x+1}=l_{x}\left( 1-q_{x} \right),$$

for age groups $x=0, 1-4, 5-9, \ldots100+$(in years). Using $l_{x}$ and $a_{x}$ values,

$$L_{x}=w_{x}l_{x+1}+a_{x}d_{x}=w_{x}l_{x+1}+a_{x}l_{x}q_{x},$$

$$T_{x}=\sum_{i=x}^{100+} L_{i},$$

where $L_{x}$ is the person-years spent in age group $x$, $d_{x}=l_{x}q_{x}$ is the number of deaths in age group $x$, and $T_{x}$ is the person-years of life remaining for those in age group $x$. The life expectancy of age group $x$, $e_{x}$, is then calculated as:

$$e_{x}=\frac{T_{x}}{l_{x}},$$

and the life expectancy at birth is calculated as $e_{0}=T_{0}/l_{0}$. Note that in our calculation of both abridged and complete life tables, we used the same $a_{x}$ values as provided by JMD (based on [5]) for each year except for the oldest age group, for which we followed the convention $a_{\infty}=1/m_{\infty}$.

For the age- and cause-specific decomposition of life expectancy change, we used the Arriaga method for age- and cause-specific decomposition of life expectancy change. [4,6,7] The total contribution of age group $x$ to the life expectancy change (in years), denoted as $C_{x}$, can be described as:

$C_{x}= \left[ \frac{l_{x}^{2020}}{l_{0}} \left( \frac{L_{x}^{2021}}{l_{x}^{2021}}-\frac{L_{x}^{2020}}{l_{x}^{2020}} \right) \right]+ \left[ \frac{T_{x+1}^{2021}}{l_{0}}\left( \frac{l_{x}^{2020}}{l_{x}^{2021}}-\frac{l_{x+1}^{2020}}{l_{x+1}^{2021}} \right) \right].$

We then decomposed $C_{x}$ into cause-specific contributions:

$C_{x}^{i}=C_{x}\left[ \frac{R_{x}^{i,2021}m_{x}^{2021}-R_{x}^{i,2020}m_{x}^{2020}}{m_{x}^{2021} - m_{x}^{2020}} \right]$,

where $C_{x}^{i}$ is the contribution of cause of death $i$ in age group $x$_,_ $R_{x}^{i}$ is the proportion of deaths in age group $x$associated with cause $i$, and $m_{x}$is the overall mortality rate in age group $x$.

**References**

1. Preston S, Heuveline P, Guillot M. Demography. Chichester, England: Wiley-Blackwell; 2000.

2. Chiang CL. The life table and its applications. Melbourne, FL: Krieger Publishing Company; 1984.

3. Keyfitz N, Caswell H. Applied mathematical demography. 3rd ed. New York, NY: Springer; 2005.

4. Munira MS, Okada Y, Nishiura H. Life-expectancy changes during the COVID-19 pandemic from 2019-2021: estimates from Japan, a country with low pandemic impact. PeerJ. 2023;11: e15784.

5. Ishii F. Construction of the Japanese Mortality Database (in Japanese). Jinko Mondai Kenkyu. 2015/3;71: 3–27.

6. Arriaga EE. Measuring and explaining the change in life expectancies. Demography. 1984;21: 83–96.

7. Arriaga EE. Changing trends in mortality decline during the last decades. Differential Mortality. Oxford University PressOxford; 1995. pp. 105–129.
