# Supplementary material for: Changes in life expectancy and life span equality during the COVID-19 epidemic in 2020-22 in Japan
Source: PLoS One. 2026 Apr 29;21(4):e0345579. doi: 10.1371/journal.pone.0345579 (PMC13134763; doi:10.1371/journal.pone.0345579)
Supplement: S2 Methods — (DOCX) [file pone.0345579.s003.docx]

**S2 Methods: Statistical details and additional analyses for the prefectural analysis of the relationship between COVID-19 statistics and life expectancy changes.**

**Heteroscedasticity and non-normality**

As described in the main text, we conducted ordinary least square (OLS) regression with linear terms of covariates in our analyses as follows:

$$\Delta e_{0}\sim\beta_{0}+\beta_{1}\log\left( X \right),$$

Where $X$ stands for covariates. Given that the sample size (n=47, which is the number of prefectures in Japan) is small, and that the residuals in OLS regression showed heteroscedasticity and non-normality in some analyses(S1 Table), standard errors were computed using the HC3 estimator [1,2] with further validation by wild bootstrap inference [3] (comparison of results from HC3 and wild bootstrap inference are shown in S2 Table)

**Linearity assumption**

In addition to the OLS regression with linear terms for covariates, we also tested the model with additional quadratic terms as follows:

$$\Delta e_{0}\sim\beta_{0}+\beta_{1}\log\left( X \right)+\beta_{2}\left( \log\left( X \right) \right)^{2}.$$

We compared linear and quadratic specifications using the robust Wald test based on HC3 covariance matrix estimator. (result in S3 Table)

**Year-to-year difference in slopes**

To explicitly obtain estimates on the year-to-year difference in intercepts and slopes for covariates, we also conducted OLS regression based on the following model:

$$\Delta e_{0}\sim\beta_{0}+\beta_{1}\boldsymbol{1}_{\boldsymbol{2021-22}}+\left( \beta_{2}+\beta_{3}\boldsymbol{1}_{\boldsymbol{2021-22}} \right)\log\left( X \right).$$

The results did not reveal statistically significant difference in slopes between 2020-21 and 2021-22 in all analyses. (result can be found in S4 Table)

**References**

1. Zeileis A. Econometric computing with HC and HAC covariance matrix estimators. J Stat Softw. 2004;11: 1–17.

2. MacKinnon JG, White H. Some heteroskedasticity-consistent covariance matrix estimators with improved finite sample properties. J Econom. 1985;29: 305–325.

3. Davidson R, Flachaire E. The wild bootstrap, tamed at last. J Econom. 2008;146: 162–169.
