# Supplementary material for: Changes in life expectancy and life span equality during the COVID-19 epidemic in 2020-22 in Japan
Source: PLoS One. 2026 Apr 29;21(4):e0345579. doi: 10.1371/journal.pone.0345579 (PMC13134763; doi:10.1371/journal.pone.0345579)
Supplement: S3 Methods — (DOCX) [file pone.0345579.s004.docx]

**S3 Methods: Numerical details of the decomposition of changes in life span equality** $h$ **and related demographic indicators.**

The decomposition of $\Delta\log\left( e_{0} \right)$, $\Delta\log\left( e^{\dagger} \right)$, $\Delta e^{\dagger}$, and $\Delta h$ as described in section 2.4 in the main text were implemented using a discretization of the path integral and a central finite-difference approximation of the partial derivatives along the path. [1]

**Decomposition framework**

Here we briefly describe the decomposition framework applied to $\Delta h$ as an example. By assuming a linearized path for the change in age- and cause-specific mortality, at time $t=t_{1}+\theta\left( t_{2}-t_{1} \right) , t_{2}>t_{1}, \theta\in\left[ 0, 1 \right]$, the mortality rate in age $x$ by cause $c$ can be described as $m_{x, c}\left( t \right)= m_{x, c}\left( t_{1} \right)+\alpha_{x, c}\left( m_{x, c}\left( t_{2} \right)-m_{x, c}\left( t_{1} \right) \right)$. Here we naturally assume $\alpha_{x, c}=\theta$ for all age $x$ and cause $c$. Thus, the path integral can be written as the sum of changes in $h$ due to each cause on each point:

$$\Delta h=h\left( \boldsymbol{t}_{\boldsymbol{2}} \right)-h\left( \boldsymbol{t}_{\boldsymbol{1}} \right)=\int_{t_{1}}^{t_{2}} \sum_{x, c} \left( \left. \frac{\partial h}{\partial\alpha_{x, c}} \right|_{t}\cdot\frac{d\alpha_{x, c}}{dt} \right)dt=\sum_{x, c} \int_{0}^{1} \left. \frac{\partial h}{\partial\alpha_{x, c}} \right|_{t=t_{1}+\theta\left( t_{2}-t_{1} \right)}d\theta\equiv\sum_{x, c} \Delta h_{x, c},$$

We numerically calculated $\Delta h_{x, c}$ for age 0 to 110+ and the 10 major death causes in 2019-20, 2020-21, and 2021-22 for the total population of Japan.

**Path discretization and central-difference approximation of partial derivatives**

We firstly set the increment of $\theta$ as $\Delta\theta=\frac{1}{N}$, where $N$ is the number of increments for numerical approximation. Then, in the case of $\Delta h$, approximation of the path integral assuming a linear mortality change during the interval of interest reduces to evaluating

$$\left. \frac{\partial h}{\partial\alpha_{x, c}} \right|_{t}=\left. \frac{\partial\log\left( e_{0} \right)}{\partial\alpha_{x, c}} \right|_{t}-\left. \frac{\partial\log\left( e^{\dagger} \right)}{\partial\alpha_{x, c}} \right|_{t}$$

at $t_{k}=t_{1}+\theta_{k}\left( t_{2}-t_{1} \right)$ for all $x, c$ where $\theta_{k}=\frac{k-0.5}{N}, k=1, 2, \ldots, N$. Terms $\left. \frac{\partial\log\left( e_{0} \right)}{\partial\alpha_{x, c}} \right|_{t}$ and $\left. \frac{\partial\log\left( e^{\dagger} \right)}{\partial\alpha_{x, c}} \right|_{t}$ were approximated as follows. For a mortality matrix $M\left( t_{k} \right)=\left( m_{x, c}\left( t_{k} \right) \right)$ at time $t_{k}$, we calculated $e_{0}$ and $e^{\dagger}$ for $M_{x, c}^{Fwd}\left( t_{k} \right)=M\left( t_{k} \right)+\frac{\Delta\theta}{2}E_{x, c}dM_{x,c}$ and $M_{x, c}^{Bwd}\left( t_{k} \right)=M\left( t_{k} \right)-\frac{\Delta\theta}{2}E_{x, c}dM_{x,c}$, where $E_{x, c}$ is an indicator matrix at element $\left( x, c \right)$, and $dM_{x,c}=M_{x,c}\left( t_{2} \right)-M_{x,c}\left( t_{1} \right)$. Then, we calculated

$$\left. \Delta{\log\left( e_{0} \right)}_{x, c} \right|_{t_{k}}=\log\left( e_{0}\left( M_{x, c}^{Fwd}\left( t_{k} \right) \right) \right)-\log\left( e_{0}\left( M_{x, c}^{Bwd\left( t_{k} \right)} \right) \right),$$

$$\left. \Delta{\log\left( e^{\dagger} \right)}_{x, c} \right|_{t_{k}}=\log\left( e^{\dagger}\left( M_{x, c}^{Fwd}\left( t_{k} \right) \right) \right)-\log\left( e^{\dagger}\left( M_{x, c}^{Bwd}\left( t_{k} \right) \right) \right),$$

$$\left. \Delta{\log\left( h \right)}_{x, c} \right|_{t_{k}}=\left. \Delta{\log\left( e_{0} \right)}_{x, c} \right|_{t_{k}}-\left. \Delta{\log\left( e^{\dagger} \right)}_{x, c} \right|_{t_{k}}.$$

Note that $e_{0}$ and $e^{\dagger}$ along the path were obtained from calculation of the complete life table with the procedures as described in S1 Methods. As for $a_{x}$ values required for life table calculation, we did not apply any interpolation because

- year-on-year difference of $a_{0}$ provided by JMD was at most 0.01 during 2000-22. Therefore, we simply set $a_{0}$ to the same value as the starting year of decomposition
- $a_{110+}$ can be calculated at each point on the path following the convention $a_{110+}=1/m_{110+}$
- $a_{x}$ values other than $a_{0}$ and $a_{110+}$ from JMD were all $0.5$.

**Numerical consistency check**

For $\Delta e^{\dagger}$, $\Delta\log\left( e_{0} \right)$, and $\Delta\log\left( e^{\dagger} \right)$, we validated our numerical approximation by assessing

1. Endpoint balance: comparison between the sum of Horiuchi contributions with the change between the two endpoints; relative error < 1e−5.
2. Convergence with respect to the path discretization parameter $N$ by comparing results computed with $N$ (we chose 10) and $2N$: relative error < 1e−5 of Frobenius norm for age×cause contribution matrices and L2 norm for age-only contribution vectors.

**References**

1. Horiuchi S, Wilmoth JR, Pletcher SD. A decomposition method based on a model of continuous change. Demography. 2008;45: 785–801.
