# Supplementary material for: Changes in life expectancy and life span equality during the COVID-19 epidemic in 2020-22 in Japan
Source: PLoS One. 2026 Apr 29;21(4):e0345579. doi: 10.1371/journal.pone.0345579 (PMC13134763; doi:10.1371/journal.pone.0345579)
Supplement: S4 Methods — (DOCX) [file pone.0345579.s005.docx]

**S4 Methods: Changes in life span equality and its sensitivity to changes in mortality.**

We used a complete (1×1 year) life table provided by JMD to calculate $h\left( t \right)=-\log\left( \bar{H}\left( t \right) \right)$ from 2000 to 2022 for total, male, and female populations, and evaluated the relationship between $h(t)$ and life expectancy at birth, $e_{0}\left( t \right)$, for each of these populations. [1] Aburto et al. [2] described the variation of $h(t)$ over time as:

$$\frac{\partial h}{\partial t}=-\frac{\frac{\partial\bar{H}}{\partial t}}{\bar{H}}=\int_{0}^{\infty} w\left( x,t \right)W_{h}\left( x,t \right)\rho\left( x,t \right)dx,$$

where

$$\rho\left( x,t \right)=-\frac{\partial}{\partial t}\log\left( \mu\left( x,t \right) \right)$$

is the mortality improvement in age $x$ over time,

$$w\left( x,t \right)=\mu\left( x,t \right)l\left( x,t \right)e\left( x,t \right)=d\left( x,t \right)e\left( x,t \right)$$

is the weight of the contribution of $\rho\left( x,t \right)$ to life expectancy change in age $x$, and

$$W_{h}\left( x,t \right)=\frac{1}{e_{0}}-\frac{1}{e^{\dagger}}\left( H\left( x,t \right)+\bar{H}\left( x,t \right)-1 \right)=\frac{1}{e_{0}}-\frac{1}{e^{\dagger}}\left( \int_{0}^{x} \mu\left( x,t \right)dx+\frac{e^{\dagger}\left( x,t \right)}{e\left( x,t \right)}-1 \right).$$

Thus, $w\left( x,t \right)W_{h}\left( x,t \right)$ can be considered as the weight, or sensitivity, of $h(t)$ to the rate of mortality improvement $\rho\left( x,t \right)$. We also calculated $w\left( x,t \right)W_{h}\left( x,t \right)$ and its threshold age $a^{H}$ that satisfies $W_{h}\left( a^{H},t \right)=0$ for $t=2000, 2001, \ldots, 2022$. These results were compared with year-on-year mortality improvement, i.e., $r\left( x, t \right)=\log\left( \mu\left( x, t \right) \right)-\log\left( \mu\left( x, t+1 \right) \right)$, which is analogous to the rate of mortality improvement as described above.

The results are shown in S13-S15 Fig and S1 Data.

**References**

1. National Institute of Population and Social Security Research. The Japanese Mortality Database. 5 Dec 2023 [cited 24 Oct 2024]. Available: https://www.ipss.go.jp/p-toukei/JMD/index-en.asp

2. Aburto JM, Villavicencio F, Basellini U, Kjærgaard S, Vaupel JW. Dynamics of life expectancy and life span equality. Proc Natl Acad Sci U S A. 2020;117: 5250–5259.
