# Supplementary material for: Changes in life expectancy and life span equality during the COVID-19 epidemic in 2020-22 in Japan
Source: PLoS One. 2026 Apr 29;21(4):e0345579. doi: 10.1371/journal.pone.0345579 (PMC13134763; doi:10.1371/journal.pone.0345579)
Supplement: S1 Table — (DOCX) [file pone.0345579.s021.docx]

**S1 Table. Prefectural linear regression analysis, residual diagnostics (Shapiro–Wilk and Breusch–Pagan tests)**

| COVID-19 indicator  (per 100k) | Period | Shapiro W | Shapiro p | BP statistic | BP df | BP p |
| --- | --- | --- | --- | --- | --- | --- |
| Cases | 2020–21 | 0.977 | 0.486 | 3.617 | 1 | 0.057 |
|  | 2021–22 | 0.969 | 0.239 | 2.194 | 1 | 0.139 |
| ICU person-days | 2020–21 | 0.971 | 0.285 | 4.211 | 1 | 0.040 |
|  | 2021–22 | 0.951 | 0.046 | 3.053 | 1 | 0.081 |
| COVID-19 deaths | 2020–21 | 0.976 | 0.451 | 6.077 | 1 | 0.014 |
|  | 2021–22 | 0.974 | 0.381 | 0.003 | 1 | 0.954 |
