# Supplementary material for: Changes in life expectancy and life span equality during the COVID-19 epidemic in 2020-22 in Japan
Source: PLoS One. 2026 Apr 29;21(4):e0345579. doi: 10.1371/journal.pone.0345579 (PMC13134763; doi:10.1371/journal.pone.0345579)
Supplement: S2 Table — (DOCX) [file pone.0345579.s022.docx]

**S2 Table. Prefectural linear regression analysis, validation of confidence interval estimates by wild bootstrap method.**

| COVID-19 indicator (per 100k) | Period | Term | Method | Estimate (95% CI) | Std. error | p |
| --- | --- | --- | --- | --- | --- | --- |
| Cases | 2020–21 | Intercept | HC3 | 0.534 (-0.137, 1.205) | 0.342 | 0.119 |
|  |  |  | Wild bootstrap | 0.534 (-0.081, 1.149) | 0.314 | 0.096 |
|  |  | Slope | HC3 | -0.104 (-0.204, -0.003) | 0.051 | 0.043 |
|  |  |  | Wild bootstrap | -0.104 (-0.195, -0.012) | 0.047 | 0.031 |
|  | 2021–22 | Intercept | HC3 | 0.534 (-5.383, 6.451) | 3.019 | 0.860 |
|  |  |  | Wild bootstrap | 0.534 (-4.476, 5.544) | 2.556 | 0.835 |
|  |  | Slope | HC3 | -0.103 (-0.696, 0.490) | 0.303 | 0.734 |
|  |  |  | Wild bootstrap | -0.103 (-0.605, 0.399) | 0.256 | 0.690 |
| ICU person-days | 2020–21 | Intercept | HC3 | 0.239 (-0.127, 0.604) | 0.186 | 0.200 |
|  |  |  | Wild bootstrap | 0.239 (-0.098, 0.576) | 0.172 | 0.172 |
|  |  | Slope | HC3 | -0.082 (-0.156, -0.009) | 0.038 | 0.029 |
|  |  |  | Wild bootstrap | -0.082 (-0.151, -0.014) | 0.035 | 0.023 |
|  | 2021–22 | Intercept | HC3 | -0.272 (-0.609, 0.065) | 0.172 | 0.114 |
|  |  |  | Wild bootstrap | -0.272 (-0.578, 0.033) | 0.156 | 0.088 |
|  |  | Slope | HC3 | -0.052 (-0.128, 0.023) | 0.038 | 0.173 |
|  |  |  | Wild bootstrap | -0.052 (-0.120, 0.015) | 0.034 | 0.135 |
| COVID-19 deaths | 2020–21 | Intercept | HC3 | -0.020 (-0.245, 0.205) | 0.115 | 0.860 |
|  |  |  | Wild bootstrap | -0.020 (-0.215, 0.174) | 0.099 | 0.840 |
|  |  | Slope | HC3 | -0.067 (-0.176, 0.042) | 0.056 | 0.226 |
|  |  |  | Wild bootstrap | -0.067 (-0.161, 0.026) | 0.048 | 0.166 |
|  | 2021–22 | Intercept | HC3 | -0.125 (-0.675, 0.425) | 0.281 | 0.656 |
|  |  |  | Wild bootstrap | -0.125 (-0.591, 0.341) | 0.238 | 0.602 |
|  |  | Slope | HC3 | -0.107 (-0.266, 0.053) | 0.081 | 0.191 |
|  |  |  | Wild bootstrap | -0.107 (-0.242, 0.029) | 0.069 | 0.131 |
