# Supplementary material for: Changes in life expectancy and life span equality during the COVID-19 epidemic in 2020-22 in Japan
Source: PLoS One. 2026 Apr 29;21(4):e0345579. doi: 10.1371/journal.pone.0345579 (PMC13134763; doi:10.1371/journal.pone.0345579)
Supplement: S3 Table — (DOCX) [file pone.0345579.s023.docx]

**S3 Table. Prefectural linear regression analysis, comparison between linear and quadratic model.**

| COVID-19 indicator (per 100k) | Period | Wald χ² | df | p |
| --- | --- | --- | --- | --- |
| Cases | 2020–21 | 0.377 | 1 | 0.539 |
|  | 2021–22 | 1.199 | 1 | 0.274 |
| ICU person-days | 2020–21 | 0.456 | 1 | 0.500 |
|  | 2021–22 | 1.635 | 1 | 0.201 |
| COVID-19 deaths | 2020–21 | 0.707 | 1 | 0.400 |
|  | 2021–22 | 0.000 | 1 | 0.987 |
