# Supplementary material for: Changes in life expectancy and life span equality during the COVID-19 epidemic in 2020-22 in Japan
Source: PLoS One. 2026 Apr 29;21(4):e0345579. doi: 10.1371/journal.pone.0345579 (PMC13134763; doi:10.1371/journal.pone.0345579)
Supplement: S4 Table — (DOCX) [file pone.0345579.s024.docx]

**S4 Table. Prefectural linear regression analysis, slope change from 2020-21 to 2021-22.**

| COVID-19 indicator (per 100k) | Slope Change (95% CI) | p (HC3) |
| --- | --- | --- |
| Cases | 0.001 (-0.600, 0.603) | 0.997 |
| ICU person-days | 0.030 (-0.075, 0.136) | 0.576 |
| COVID-19 deaths | -0.039 (-0.233, 0.154) | 0.690 |
