# Supplementary material for: Changes in life expectancy and life span equality during the COVID-19 epidemic in 2020-22 in Japan
Source: PLoS One. 2026 Apr 29;21(4):e0345579. doi: 10.1371/journal.pone.0345579 (PMC13134763; doi:10.1371/journal.pone.0345579)
Supplement: S5 Table — (DOCX) [file pone.0345579.s025.docx]

**S5 Table. Trend of death attributable to senility from 2019 to 2022.**

| Year | All cause | Other causes | Senility | % of Senility in other causes |
| --- | --- | --- | --- | --- |
| 2019 | 1,381,093 | 212,426 | 121,863 | 57.4 |
| 2020 | 1,372,755 | 222,549 | 132,440 | 59.5 |
| 2021 | 1,439,856 | 245,721 | 152,027 | 61.9 |
| 2022 | 1,569,050 | 283,516 | 179,529 | 63.3 |

# original data: death counts by cause of death in Japan, from vital statistics of Japan [1]

(relevant data in S1 Data)

1. Ministry of Health Labour and Welfare. Ministry of Health, Labour and Welfare. 2024 [cited 2 Jan 2026]. Available: https://www.mhlw.go.jp/english/database/db-hw/vs01.html
