# Supplementary material for: Changes in life expectancy and life span equality during the COVID-19 epidemic in 2020-22 in Japan
Source: PLoS One. 2026 Apr 29;21(4):e0345579. doi: 10.1371/journal.pone.0345579 (PMC13134763; doi:10.1371/journal.pone.0345579)
Supplement: S6 Table — (DOCX) [file pone.0345579.s026.docx]

**S6 Table. Change in deaths per 100k population due to COVID-19, suicide, and remaining causes in those aged 10-44 in 2019-20.**

| Age group | All cause | COVID-19 | Suicide | Remaining |
| --- | --- | --- | --- | --- |
| 10-14 | 0.03 | 0.00 | 0.61 | -0.58 |
| 15-19 | 1.85 | 0.00 | 1.55 | 0.30 |
| 20-24 | 2.57 | 0.00 | 3.55 | -0.98 |
| 25-29 | 3.18 | 0.17 | 3.04 | 0.12 |
| 30-34 | 0.15 | 0.03 | 1.25 | -1.13 |
| 35-39 | 0.05 | 0.04 | 0.83 | -0.82 |
| 40-44 | 1.97 | 0.10 | 1.65 | 0.22 |

#per 100k death rate was calculated using:

-death counts by cause of death in Japan, from vital statistics of Japan [1]

-exposure to risk population for age groups, provided by JMD [2]

, which can be also found in S1 Data.

1. Ministry of Health Labour and Welfare. Ministry of Health, Labour and Welfare. 2024 [cited 2 Jan 2026]. Available: https://www.mhlw.go.jp/english/database/db-hw/vs01.html

2. National Institute of Population and Social Security Research. The Japanese Mortality Database | National Institute of Population and Social Security Research. 2023. Available: https://www.ipss.go.jp/p-toukei/JMD/index-en.asp
