# Supplementary material for: Changes in life expectancy and life span equality during the COVID-19 epidemic in 2020-22 in Japan
Source: PLoS One. 2026 Apr 29;21(4):e0345579. doi: 10.1371/journal.pone.0345579 (PMC13134763; doi:10.1371/journal.pone.0345579)
Supplement: Attachment — Submitted filename: okada_PONE_rebuttal.docx [file pone.0345579.s028.docx]

[Point-by-point responses to Reviewers] Changes in life expectancy and life span equality during the COVID-19 epidemic in 2020-22 in Japan (Previously “Changes in life expectancy and life span equality during the COVID-19 epidemic in Japan up to 2022.”) (submitted to PLOS One)

[Response to Reviewer 1]

We appreciate your thorough and very helpful review. Please find below the point-by-point response to your comment.

1) The Introduction and Discussion sections do not mention some relevant studies on the COVID-related mortality elevation in Japan. Namely, these are:

- Hirokazu Tanaka, Shuhei Nomura, Kota Katanoda, Changes in Mortality During the COVID-19 Pandemic in Japan: Descriptive Analysis of National Health Statistics up to 2022, Journal of Epidemiology, 2025, Volume 35, Issue 3, Pages 154-159
- Tanaka H, Togawa K, Katanoda K. Impact of the COVID-19 pandemic on mortality trends in Japan: a reversal in 2021? A descriptive analysis of national mortality data, 1995–2021. BMJ Open 2023;13:e071785. doi:10.1136/ bmjopen-2023-071785
- Z Shervani,AA Khan, I Khan, A Sherwani, PDM Kumar et al. Marginal Shortening of Life Expectancy in Japan During COVID-19: A Low Pandemic Impact Country Due to Improved Health Infrastructure and Awareness. (2024). European Journal of Medical and Health Sciences, 6(6), 9-17. <https://doi.org/10.24018/ejmed.2024.6.6.2214>
- Zameer Shervani, Aamir Akbar Khan, Intazam Khan et al. Factors Explaining Japan’s Low COVID-19 Mortality: Comparison with Rich and Democratic Countries. (2025). European Journal of Medical and Health Sciences, 7(1), 1-9. <https://doi.org/10.24018/ejmed.2025.7.1.2245>
- Mst Sirajum Munira, Okada Y, Nishiura H. 2023. Life-expectancy changes during the COVID-19 pandemic from 2019–2021: estimates from Japan, a country with low pandemic impact. PeerJ 11:e15784 DOI 10.7717/peerj.15784

These recent studies are thematically close to the present study. They should be mentioned in the Introduction and/or Discussion.

Similarities or differences between the present study and the earlier studies should be noted. The readers should know what novelty the present study adds to the existing literature.

>>

Response:

We agree that these references should be referred to in the manuscript. We added the suggested references as suggested, with relevant descriptions added to the original manuscript. (L82-83, L85-91, L363-366, L375-L377)

2) The study focuses on annual changes in e0 in 2020-22. However, the full amount of e0 losses in these years can be assessed only through comparisons between the observed e0 values and the counterfactual (predicted) e0 values. Using the Human Mortality Database (HMD) series for Japan, one can see that from 2012 to 2019, the Japanese life expectancy was increasing by 0.18 years per calendar year on average. If we apply this rate of increase to 2020-22, the e0 losses for Japan in 2021 and 2022 would be 0.38 and 0.98 years instead of the observed (annual declines) of 0.13 and 0.48 years.

This difference is important and should be acknowledged. It would also be interesting to see speculations about possible differences between the annual decreases in e0 in 2020-22 and the e0 losses in the same years.

>>

Response:

We agree that we should have added counterfactual e0 values for 2020-22. We added the comparison of actual (observed) life expectancy and a counterfactual LE (10-year-average change from 2010-2019 is maintained up to 2022) to the new “Table 1” in the revised manuscript (former S1 Table)

3) The manuscript has a massive Methods section. It is not clear whether it is a methodology paper or a substantive analysis. There are numerous formulae. All of them are known from earlier studies. The first seven equations are completely unnecessary, as they are very conventional and can be found in demography textbooks, such as those by Preston or Keyfitz, or Chiang.

>>

Response:

We agree that the original manuscript included conventional methodologies which can be spared in the main text. We moved such descriptions to the supplement, and added relevant references to the main text as suggested. (L 105-114, S1 Methods)

4) The following equations for the analysis of the age at death equality h are less trivial since they are newer. Maybe the formal definitions of the entropy H, e+ (e-dagger), and the equality h could remain in the main text. The next four equations related to further analysis of temporal changes in h should be given in a supplementary appendix.

Instead, the reader needs a transparent explanation of all measures and calculation procedures. The substantive sense of h should be clarified. Without a transparent explanation, it's impossible to understand why h is used and what is the public health sense of its decrease by 0.07 between 2019 and 2020.

Perhaps it would be better to use e+ instead of h. e+ is a transparent measure of life expectancy losses by Vaupel and Canudas-Romo. e+ is counted in years of life and has a clear public health sense (Shkolnikov et al. 2011).

Shkolnikov V.M., Andreev E.M., Zhang Z., Oeppen J., J.W.Vaupel. 2011. Losses of expected lifetime in the United States and other developed countries: methods and empirical analyses. Demography, 48: 211-239

>>

Response:

a) Equations

We moved four equations about the temporal changes in h to S4 Methods.

b) Explanation on measures and calculation procedures

We agree that the interpretation of h and e+ should have been clearly stated. To address this issue, we added descriptions

-to that help readers understand e+(reference added), and the difference between e0, e+, h, H (L132-138)

-to help interpret changes in H or h = -log(H) (L120~ L130)

c) Choice of metric to evaluate life span equality or life disparity

We clarified the merit of evaluating h in L139-145, with an intuitive example to guide interpretation. We also agree that e+ is a measure that should be highlighted in the main analysis. Therefore, in the result section we also

-changed panel (B) in Fig 4 to a 2D plot showing the historical relationship between $\Delta\log\left( e_{0} \right)$ and $\Delta\log\left( e^{\dagger} \right)$, so that what we described in L139-145 links to the results.

-added S10-S12 Fig that shows the decomposition results of the yearly changes of e+ by age and cause from 2019-20 to 2021-22, which helps understand the relationship between h and e+, with relevant descriptions in the result section. (L288-L291)

5) Despite the length of the Methods, they lack a precise description of the data and computational procedures. Which calculations were based on the complete life tables vs. the abridged life tables? It is clearly preferable to use the single-year-age data for calculating dispersion measures such as h or e+. This information is needed. It can be provided in a supplementary appendix.

>>

Response:

We should have provided precise information on this issue.

For the calculation of h and e+, we used the complete life tables provided by JMD. For prefectural analysis and Arriaga decomposition, we used the abridged life tables we re-calculated based on the abridged life tables provided in JMD

We added relevant explanation. (L105- L114, L130)

6) The analysis of causes of the h changes between 2019 and 2020, 2020 and 2021, 2021 and 2022 is not convincing. From the methodology point of view, it is possible to see directly the components of changes in h. This could be done by decomposing the change in h by age and CODs with one of the universal decomposition methods (discrete replacement method by Andreev et al. 2002; continuous change method by Horiuchi et al. 2008). These methods estimate additive contributions of different ages and causes of death to a change in any aggregate measure. This has not been done. Looking at changes in M(x) or at the weights W(x) cannot replace the direct decomposition.

On the substantive side, there are also some problems. First, the alarming drop in h between 2019 and 2020 is not explained. However, Figure 3 reveals moderate mortality increases at young adult ages in 2020. My own quick look at the Human Mortality Database data for Japan also indicates mortality increases at ages between 20 and 29 as well as some ages between 30 and 44 in 2020. So, what happened in 2020? It is important to know. The potential role of the pandemic is doubtful. Second, increases in h from 2020 to 2021 and from 2021 to 2022 are considered “undesirable” as they were (likely) caused by the mortality elevation at old and very old ages. At the same time, the h increase in 2021-22 may be considered an advantage of Japan compared to other countries. While in Japan, the mortality increase in 2021 and especially in 2022 was entirely concentrated at old ages, in many other countries (USA, Eastern Europe, Britain, Belgium, and other), the COVID-related mortality increases were also large at midlife and “young old” ages (55-69). The latter pattern results in stagnant or even decreasing values of h.

That is to say that the changes in e0 and e+ in Japan should be considered in context of e0 and e+ changes elsewhere.

>>

Response:

Thank you very much for your very important suggestion.

a) Decomposition of h by age and cause of deaths

We conducted decomposition by applying the approach of Horiuchi et al as suggested. The relevant methodologies were added in the methods section (L138-L146, S3 Methods), and the results are presented in Fig 5 and in S1 Data. (We also provided the same decomposition results for e+ in S10-S12 Fig.)

b) Mortality increase in young adults in 2020:

We should have provided more comprehensive explanation for this. Suicide as an important cause of death that elevated mortality in 2020 among young adults in Japan has been revealed in published studies. Together with these preceding studies, we also added a supplementary analysis (S6 Table) to clarify the impact of suicide in those aged 10-44 in 2020. This is mentioned in the relevant part of the discussion section. (L363-368)

c) Further interpretation of “h” from 2020-22.

We agree that “increase in h” may not necessarily be an “undesirable” consequence as we argued in the original text, without further analyses.

To address this issue, firstly, we added a new figure panel in Fig 4 that compares $\Delta\log\left( e_{0} \right)$ against $\Delta\log\left( e^{\dagger} \right)$.

This revealed that, by visual inspection, 2019-20 was an outlier in that $\Delta\log\left( e^{\dagger} \right)$ increased substantially while positive $\Delta\log\left( e_{0} \right)$ was maintained. However, after 2019-20, it turned out that both $\Delta\log\left( e_{0} \right)$ and $\Delta\log\left( e^{\dagger} \right)$ turned negative and grew larger in magnitude up to 2021-22.

We also placed Fig 5 that presents the results of decomposition of $\Delta h$ by age and cause in 2019-20, 2020-21, 2021-22, which now clearly describes the contribution of elderly deaths as the leading cause of “increase in h” in 2020-21 and 2021-22, whereas contributions from younger adults were overall negative to neutral overall.

7) Discussion about the large increases in mortality from CVD in 2021 and 2022 does not take into account potential direct contributions of COVID-19 to deaths from CVD. COVID-19 could appear on the medical death certificate as an immediate, intermediate, or contributory cause. It would be good to discuss how large the direct influence of COVID-19 could be in CVD deaths in Japan.

>>

Response:

We regret that this issue should rather be discussed as a limitation of our study, because there is no public data to explore the possible bias regarding the death certificate. However, we may argue the potential effect of COVID-19 on CVD, given that there is a published study on age-standardized mortality rates by cause of death in Japan suggests an increase in ASMR due to “Heart diseases” that is roughly proportional to increase in ASMR due to “COVID-19”. We added relevant discussions in the revised manuscript (L341-L348)

8) The analysis of statistical associations between e0 decreases in 2021 and 2022 and the epidemiological indicators is not fully justified. The authors should perform statistical tests for normality, heteroscedasticity, and linearity. Pearson’s r values together with p-values should be shown on these graphs. The regression outcomes are commented as if there is a radical difference between 2021 and 2022. It looks like statistical links exist in 2021 and they do not exist in 2022. However, the scatterplots and the tables show that in both years, the links are very weak, but in 2022 some of them are statistically significant.

>>

Response:

We agree we should have provided richer statistical tests and consideration for normality, heteroscedasticity, and linearity (L 119-128)

a) Firstly, we added Pearson’s r values with p-values as suggested to the plots.
b) In our linear regression including only the 1^st^ order (linear) term, Shapiro-Wilk test suggested deviation from normality in residuals for the “2022 person-days in intensive care “ OLS. The Breusch-Pagan test also suggested heteroscedasticity in “2021 person-days in intensive care” and “2021 deaths”. (S1 Table)
c) To account for heteroscedasticity, we report inference based on heteroscedasticity-robust (sandwich) standard errors throughout.
d) To assess potential non-linearity, we conducted comparison between linear regression models with “1^st^ order term (assuming linearity; main analysis)” with “1^st^ + 2^nd^ (quadratic) order terms (assuming non-linearity) in this framework. Across all analyses, the quadratic term did not improve model fit and the Wald test did not support choosing quadratic models. Thus, our main analyses are based on linear specification.(S3 Table)
e) Given that non-normality was suggested in some models, we also conducted wild bootstrap inference to obtain robust standard errors for validating the results from sandwich estimators. The results from wild bootstrap were consistent with those from sandwich estimators in all analyses. (S2 Table)
f) To visualize the “slope change” from 2020-21 to 2021-22, we also conducted supplementary analyses by the following regression model: (in S2 Method)

$$\Delta e_{0}\sim\beta_{0}+\beta_{1}\boldsymbol{1}_{\boldsymbol{2021-22}}+\left( \beta_{2}+\beta_{3}\boldsymbol{1}_{\boldsymbol{2021-22}} \right)\log\left( x \right),$$

Which essentially yielded same results as in individual anaiyses but provides more clarity. These supplementary analyses revealed non-significant change in the “slopes” in all models from 2020-21 to 2021-22. Based on these results, we also revised the text to avoid overstating year-to-year differences between 2021 and 2022.(S4 Table)

9) Overall evaluation of the Japanese fight against death in 2020-22: The manuscript sounds quite alarming. However, previous studies presented Japan as a country with low/very moderate losses and as a “success story”. It would be good to address this in the Discussion.

>>

Response:

We added relevant discussion in L315-323 with additional references as suggested.

10) Language. The manuscript would benefit from its further editing by a native English speaker. For example, the title “Changes in life span equity from 2020-22 in Japan” is confusing. It would be possible to say “in 2020-22” or from “2019 to 2022”.

>>

Response:

We revised the title as suggest. We also checked our English writing again throughout the revised manuscript.

[Response to Reviewer 2]

We truly appreciate your very fundamental advice on our manuscript. Please find below the point-by-point response to your comment.

<Major>

1) Interpretation of prefectural analysis needs caution. The manuscript argues that the absence of correlation between prefectural COVID-19 statistics and life expectancy change in 2021–22 indicates “growing ascertainment bias”. With only 47 prefectures, linear regression power is limited. COVID-19 cases, ICU days, and deaths suffer from known reporting issues, but the manuscript does not quantify potential misreporting.

>>

Response:

We agree that our results only suggest correlation and the message should be about suggestions on one possible mechanism that may underly our findings. We set a lower ton on this argument in relevant sentences (L198-206, L333-338)

In addition to correcting the overstatement of our findings here, we upgraded our analysis using linear regressions as described in L119- L128 and S1-S4 Table.

2) Life expectancy changes include all-cause mortality, not just COVID-19: structural health-system strain, ageing, deferred healthcare, and influenza resurgence might all explain discrepancies. Instead of asserting ascertainment bias, acknowledge it as one possible explanation among others, and emphasize the ecological, multi-cause nature of the prefectural relationships.

>>

Response:

We agree that our assertion on ascertainment bias was too strong and we should have listed it as one among other possibilities. We revised relevant sentences as suggested. (L38, L392-L394)

3) Cause-of-death decomposition requires deeper contextualization. The decomposition clearly shows increased contributions of cardiovascular and “other” causes in 2022 (Fig. 3). However, “other causes” is too broad. Senility, accidents, metabolic disorders? If senility drives most changes, this should be explicitly shown, not only mentioned.

Cardiovascular increases are attributed to COVID-19-related indirect effects, but the manuscript does not present evidence for causal mechanisms. Add a supplemental table with the breakdown of “other causes.” Add a paragraph clarifying whether increases in cardiovascular deaths follow known international post-COVID trends or reflect Japan-specific dynamics.

>>

Response:

We agree we should have provided details supporting our argument. Thus,

-We added S5 table and Supplementary Data to clarify the magnitude of increase in senility as a driver of surge in mortality in “other” causes.

-About cardiovascular deaths, we divided the paragraph in the discussion section of the original manuscript that included descriptions of cardiovascular deaths into two new paragraphs. In the new paragraph on cardiovascular deaths, we added a few references to describe the risk of cardiovascular diseases / deaths due to COVID-19, as well as the impact of cardiovascular deaths on life expectancy changes in multinational settings during COVID-19. (L341- L348)

4) Life-span equality section is underdeveloped. The discussion of ℎ (life span equality) is technically correct but insufficient for non-specialist readers. Interpretation is vague: “undesirable increase in life span equality” (lines 314-315) may confuse readers unfamiliar with life table entropy. The explanation linking increased ℎ to higher mortality above threshold age a_h is technically valid but described in overly mathematical terms. Life span equality increased because mortality increases were concentrated at older ages, which compresses the age-at-death distribution. Provide a short, intuitive explanation of life table entropy and its link to lifespan compression.

>>

Response:

We agree that the discussion of h is insufficient and unclear. To address this issue, firstly, in the revised manuscript we emphasized the meaning of e-dagger and h including how temporal changes of these indicators can be interpreted in a demographic context. (L130- L138)

We also added an example to help readers interpret the changes in h as the change in concentration of the ages at death. (L143-145)

Secondly, we added an intuitive guidance in the methods section to help interpret the change of h in relation to wW and a_H. (L147 – L156). Regarding this issue, we also added additional analysis from decomposition of the change in h by ages and death causes during 2020-22 in the revised manuscript. (Fig 5) These new results may hopefully help intuitive understanding of how the changes in life span equality in 2021-22 were largely attributed to mortality increase in older ages.

5) The Results section sometimes repeats earlier work. Some paragraphs replicate findings already described in the authors’ 2019–2021 study (Munira et al., 2023), but without clearly distinguishing what is new in the update up to 2022.

Add a short subsection explicitly stating, What was already known from 2019–2021? What has changed in 2022? Why 2022 is important for Japan (e.g., Omicron, high vaccine coverage, healthcare saturation)? Graphical presentation could be improved.

>>

Response:

To clarify the distinction between results from our previous work and those that are original or new to our work, we added relevant texts and references. (L161-169)

About graphical presentation, we improved graphical representations in some figures:

-added correlation coefficient to Fig2

-added reader-friendly caption and footnote to the labels in Fig4

6) Fig. 4 (Life span equality). The vertical axis lacks interpretation: add labels or annotations explaining what changes of 0.02 in ℎ represent.

>>

Response:

We added label and footnote to enhance interpretation (L270)

7) Methods need more transparency. Missing clarifications. Were abridged life tables validated against official JMD single-year tables? Authors mention near-identity but show no numbers.

>>

Response:

The validation of our “recalculated” abridged life tables were conducted by comparison with “JMD’s” abridged life table. We added a renewed description on how we recalculated the abridged life tables in the method section. What we did was simply to shorten the JMD’s abridged life tables (original: up to 110+) so that the age group range and definition matches that of the death cause data (up to 100+). This manipulation was solely for the sake of conducting Arriaga Decomposition, which resulted in minor gaps (<0.05 years in life expectancy) compared with those by JMD.

We clarified these issues in the methods section, and provided e0 values from JMD life table in the supplementary data so that those can be compared with our calculation. (L109-111, S1 Data)

8) What values were used for ax in 2020–2022? JMD uses specific ax schedules that change in crisis years. Add a short appendix or supplemental note describing the ax assumptions, interpolation methods, quality checks.

>>

Response:

We used the same values of ax as provided in JMD both for complete and abridged life tables. Thus, we did not pose any additional assumptions, nor conducted interpolation or quality checks on our side.

About the complete lifetable of JMD, the ax in JMD basically follows HMD methodology except for a0 that are calculated from JMD’s original formula. This is described in <https://www.ipss.go.jp/p-toukei/JMD/briefsummary.html> and in Ishii (2015) (<https://www.ipss.go.jp/syoushika/bunken/data/pdf/20038302.pdf> : in Japanese) In a sense, the adaptive calculation algorithm adopted by JMD can work as a crisis adjustment. However, to our understanding, no drastic year-on-year change

We regret that we should have clarified the abovementioned issues. As suggested, we described the ax assumptions in S1 Methods.

9) Discussion of policy relevance could be strengthened. The manuscript ends with general statements but does not articulate the policy implications of increased cardiovascular mortality, increased senility deaths, decoupling of reported COVID-19 statistics from demographic indicators. A clearer final section would improve impact.

>>

Response:

We strengthened the policy relevance of our findings in the Discussion. Specifically, we added the following implications:

-timely demographic analysis of all-cause and cause-specific mortality will complement routine epidemiological surveillance

-maintaining access to emergency and time-sensitive healthcare (including cardiovascular care) is critical during large epidemic waves

-implementation of public health programs towards mental well-being and frailty prevention

(L333-338, L359-362, L398-403)

<Minor>

10) In the first sentence of the abstract, life expectancy is not wrongly defined. It says that “Life expectancy is a demographic measure of the death structure and its change at the population level.” But LE does not measure the death structure (whatever that means); it is one of the central measures in demography and actuarial science, expressing the average length of life that a newborn (or any person of a given age) is expected to live, if current mortality conditions were to remain constant throughout their remaining lifetime. Formally, life expectancy is a summary measure of mortality derived from a life table, which models the survival pattern of a hypothetical cohort subjected to age-specific mortality rates observed in a particular population and period.

>>

Response:

Thank you very much for your correction, we revised our description for correctness and clarity. (L23-24)

11) The objective of the manuscript “…the present study focuses on interpreting changes in the nature of mortality not only by life expectancy but also by the age distribution of mortality in Japan” is confusing by how it is written. I think the focus goes beyond that. Copy-editing is needed.

>>

Response:

We revised the relevant sentence in the abstract to clarify our key focus in the present study. (L24-27)

12) Neoplastics does not exist as a cause of death; perhaps you mean neoplasms.

>>

Response:

We carefully checked the manuscript and confirmed that “Neoplastic” is not used as a noun in the main text. (Some are used as adjectives in the figure labels)

13) In the Abstract says: “Beyond this measure that have been heavily applied…” must be “…has been heavily applied.”

>>

Response:

We appreciate your correction. We revised the text as suggested. (L24)

14) Clarify what life-span equality measures in one sentence.

>>

Response:

We clarified the meaning of life span equality in the relevant sentence as suggested (L34-36)

15) Introduction: Provide more background on Japanese COVID-19 mortality, including excess mortality patterns reported by other sources.

>>

Response:

We added information on COVID-19 or COVID-19-related mortality in Japan with references as suggested. (L68-78)

16) Methods: Add citations when introducing the formulas. Perhaps Preston, Heuveline and Guillot (2001). Actually, you could discard the explanation of the life table construction; just refer to it , for example, Preston et al. (2001).

Preston, S. H., Heuveline, P., & Guillot, M. (2001). Demography: Measuring and modeling population processes . Blackwell Publishers.

>>

Response:

We added references including Preston, Heuveline and Guillot (2001) and moved the explanation of the life table construction to the supplement. (L109, S1 Methods)

17) Methods: Clarify whether decomposition was symmetric or stepwise.

>>

Response:

We clarified in the relevant sentence that we conducted stepwise decomposition..(L111-114)

18) Results: Avoid repeating numeric results from S1 Table in multiple places streamline. You started the results by quoting it. If it is important, just include it in the text, not in the appendix.

>>

Response:

We agree that we should include S1 Table in the main text to enhance readability. We moved this table to the main text as “Table 1”.

19) References: Some citations appear duplicated or misformatted.

>>

Response:

We corrected some citations for clarity.
