# Supplementary material for: Changes in life expectancy and life span equality during the COVID-19 epidemic in 2020-22 in Japan
Source: PLoS One. 2026 Apr 29;21(4):e0345579. doi: 10.1371/journal.pone.0345579 (PMC13134763; doi:10.1371/journal.pone.0345579)
Supplement: Attachment — Submitted filename: okada_PONE_rebuttal_rev2.docx [file pone.0345579.s029.docx]

[Point-by-point responses to Reviewers] Changes in life expectancy and life span equality during the COVID-19 epidemic in 2020-22 in Japan (Previously “Changes in life expectancy and life span equality during the COVID-19 epidemic in Japan up to 2022.”) (submitted to PLOS One)

[Response to Reviewer 1]

We truly appreciate your review to improve our manuscript. Please find below the point-by-point response to your comment.

1) L63-L64. “…measurable mortality impact…” – better to say “considerable” or “substantial” impact.

>>

Response: We changed the expression as advised.

(L61)

2) L85-L86. “We also decomposed the year-on-year life expectancy change from 2019-22 …” sounds confusing. It should be “We also decomposed annual life expectancy changes in 2019-2022.”.

>>

Response: We changed to decomposed annual life expectancy changes” for clarity.

(L83)

3) L101. “The deaths counts by cause of death in Japan is only available…” It should be “The death counts by cause of death in Japan are only available…”

>>

Response: We corrected this sentence accordingly.

(L98)

4) L135. “from 2000-2022” It should be “from 2000 to 2022” or “for the period 2000-2022”

>>

Response: We corrected this sentence.

(L130)

5) L141-L143. It is still unclear why it is important (from the public health viewpoint) to know whether a relative e0 change is larger or smaller than the relative e-dagger change.

>>

Response: We added descriptions to clarify the importance of evaluating the changes of e-dagger in comparison to changes of e0. (L136-141)

6) L225-L226. It is better to say “Life expectancy changes from 2020 to 2021 and from 2021 to 2022”. The column heading “Year” is confusing. It is better to say “Period”.

>>

Response: We modified the title of Table 2 and the column heading as suggested.

(L220-222)

7) L233-L235. “… more eminent in 2021-22 than in 2020-21”. This is unclear. It would be better to say here “more eminent in the change from 2021 to 2022 than in the change from 2020 to 2021”.

>>

Response: We revised the text as suggested.

(L228-229)

8) L328. “The unclear correlation …. indicators of COVID-19…”. It is better to say “The unclear correlations across prefectures … indicators of COVID-19 …”.

>>

Response: We added “across prefectures” in the relevant sentence.

(L320)
